# Cytoplasmic TRAIL-R1 is a positive prognostic marker in PDAC

**DOI:** 10.1186/s12885-018-4688-8

**Published:** 2018-07-31

**Authors:** Jan-Paul Gundlach, Charlotte Hauser, Franka Maria Schlegel, Christine Böger, Christian Röder, Christoph Röcken, Thomas Becker, Jan-Hendrik Egberts, Holger Kalthoff, Anna Trauzold

**Affiliations:** 1 Department of General Surgery, Visceral, Thoracic, Transplantation and Pediatric Surgery, University Hospital Schleswig-Holstein (UKSH), Campus Kiel, Arnold-Heller Str. 3, Haus 18, 24105 Kiel, Germany; 2 Institute for Experimental Cancer Research, University of Kiel, Arnold-Heller Str. 3 (Haus 17), D-24105 Kiel, Germany; 3 Department of Pathology, University Hospital Schleswig-Holstein (UKSH), Campus Kiel, Arnold-Heller Str. 3, Haus 14, 24105 Kiel, Germany

**Keywords:** TRAIL-R1, Death receptor, Immunhistology, Pancreatic cancer

## Abstract

**Background:**

The death receptors TRAIL-R1 and TRAIL-R2 are frequently overexpressed in cancer and there is an emerging evidence for their important role in malignant progression, also in the case of pancreatic ductal adenocarcinoma (PDAC). In their canonical localization at the plasma membrane, TRAIL-R1/−R2 may induce cell death and/or pro-inflammatory signaling leading to cell migration, invasion and metastasis. Although, they have repeatedly been found intracellular, in the cytoplasm and in the nucleus, their functions in intracellular locations are still not well understood. Likewise, studies dealing with the prognostic relevance of TRAIL-Rs located in particular cellular compartments are very rare. For PDAC, the correlation of nuclear TRAIL-R2 with worse patients’ prognosis has been shown recently. Corresponding data on TRAIL-R1 are not available so far.

**Methods:**

In the present study we analyzed the expression of TRAIL-R1 in 106 PDACs and 28 adjacent, peritumoral non-malignant pancreatic ducts with special emphasis on its cytoplasmic and nuclear localization and correlated the immunohistochemical findings with clinico-pathological patient characteristics.

**Results:**

TRAIL-R1 was found in 93.4% of all PDAC samples. Cytoplasmic staining was present with very similar intensity in tumor and normal tissue. In contrast, nuclear TRAIL-R1 staining was significantly stronger in tumor compared to normal tissue (*p* = 0.006). Interestingly, we found that the number of cells with cytoplasmic TRAIL-R1 staining negatively correlates with tumor grading (*p* = 0.043). No such correlation could be detected for nuclear TRAIL-R1. Neither, cytoplasmic nor nuclear TRAIL-R1 staining showed a correlation with other clinico-pathological parameter such as pTNM categories. However, Kaplan-Meier analyses revealed significantly prolonged median survival of patients with positive cytoplasmic TRAIL-R1 expression in more than 80% of tumor cells compared to patients with tumors containing a smaller quantity of cells positively stained for cytoplasmic TRAIL-R1 (20 vs. 8 months; *p* = 0.004).

**Conclusion:**

Cytoplasmic TRAIL-R1 is a positive prognostic marker for patients with PDAC. Our findings indicate that loss of cytoplasmic TRAIL-R1 results in recurrent disease with more malignant phenotype thus suggesting anti-tumor activities of cytoplasmic TRAIL-R1 in PDAC.

## Background

Despite tremendous efforts in molecular and clinical oncology, pancreatic ductal adenocarcinoma (PDAC) still remains one of the deadliest cancers with a mortality rate almost equal to its incidence rate [1]. Its dismal prognosis results from the lack of early diagnostic options, its highly aggressive growth and a resistance to current radio- and chemotherapeutic treatments [2]. Thus, identification of new prognostic markers provides a strategy to uncover still unknown players driving PDAC malignancy and potentially to identify novel therapeutic targets.

TNF-related apoptosis inducing ligand (TRAIL) and its death inducing receptors TRAIL-R1 and TRAIL-R2 are promising candidates for the development of such novel targeted strategies. The rationale behind this assumption is the original observations that i) TRAIL induces apoptosis preferentially in tumor cells leaving normal healthy cells alive; ii) tumor cells usually express high levels of either TRAIL-R1 or TRAIL-R2 or both. These facts led to the development of different TRAIL formulations as well as agonistic TRAIL-R1- or TRAIL-R2-specific antibodies for treatment of human malignancies. However, relatively soon it has been recognized that many tumor cells are resistant to TRAIL induced apoptosis, the fact explaining the disappointing results from clinical trials [3]. In addition, it became evident that TRAIL-R1 and TRAIL-R2 may respond to TRAIL - apart from apoptosis induction - with activation of different non-apoptotic signal transduction pathways like NF-kB, ERK1/ERK2, JNK, Src and AKT [4], which can lead to the inhibition of apoptosis as well as to cell proliferation, migration and invasion. Most importantly, TRAIL receptor signaling may enhance cancer cell invasion and metastasis in vivo [5–7]. Thus, therapeutic concepts are needed which combine TRAIL receptor targeting agents with agents sensitizing tumor cells and reducing the unwanted, non-death-inducing signaling of the receptors. The important concern regarding TRAIL-receptor based anti-tumor therapy is also the observed preference for the usage of the particular TRAIL death receptor for the transmission of the TRAIL-mediated signaling in tumor cells. Generalized predictions on main death receptor responsibility for apoptosis induction in given cancer types are difficult. It is widely accepted that the preference for either TRAIL-R1 or TRAIL-R2 is a cell type specific feature. A comprehensive compilation of tumor cell lines together with their preferences for usage of the particular TRAIL death receptor is provided by Roosmalen et al. [8]. In PDAC cells, others and we have shown that regardless of the simultaneous presence of TRAIL-R1 and TRAIL-R2 at the cell surface, these cells use predominantly TRAIL-R1 when treated with recombinant TRAIL [9, 10]. Consequently, TRAIL-R1-targeting variants of TRAIL and agonistic TRAIL-R1 specific antibodies were expected to have higher therapeutic effects in the treatment of PDAC than they in fact do [3]. Interestingly, a recent report revealed that some PDAC cell lines show preference for TRAIL-R2 in inducing cell death [11] pointing to an unexpected high diversity of TRAIL receptor preference even in the same tumor entity.

Of note, under physiological conditions TRAIL has been shown to be an important effector molecule in the tumor immunosurveillance [12, 13]. On the other hand, malignant cells themselves can produce TRAIL and this may lead to an increase of their invasive and migratory properties [7]. Thus, the expression levels of TRAIL receptors as well as the preference for the usage of TRAIL-R1 or TRAIL-R2 in TRAIL-induced apoptotic/non-apoptotic signaling may be an essential factor determining both, the tumor initiation and progression.

The biological responses to TRAIL are attributed to the function of TRAIL receptors at the plasma membrane. Interestingly, although the intracellular presence of TRAIL-R1 and/or TRAIL-R2 has repeatedly been noticed, only recently the question of biological relevance and eventually specific functions of intracellular receptors began to be addressed. Obviously, sequestration of the receptors in the cytoplasm or in the nucleus, frequently observed in cancer, could represent one of the strategies used by these cells to escape TRAIL-induced apoptosis. Indeed, such mechanisms have been proposed for both cytoplasmic [14–16] and nuclear TRAIL receptors [16–18]. More recently, specific function of nuclear TRAIL-R2 has also been uncovered [19]. In the nucleus, TRAIL-R2 interacts with the microprocessor complex and impairs the maturation of the miRNA let-7. This leads to the increased levels of the malignancy promoting factors HMGA2 and Lin28B and enhances tumor cell proliferation in vitro and in vivo [19]. Likewise, specific functions of cytoplasmic TRAIL death receptors have also been proposed lately. Concrete, in response to endoplasmic reticulum (ER) stress, as a part of unfolded protein response (UPR), cytoplasmic TRAIL-R1 and TRAIL-R2 both are able to aggregate and induce cell death [20, 21].

Interestingly, although numbers of immunohistological studies addressed the issue of the impact of differentially expressed TRAIL death receptors in tumor and corresponding normal healthy tissue, the clinical relevance of TRAIL receptors present in particular intracellular compartment, cytoplasm or nucleus was analyzed only sporadically. For PDAC we reported recently that high levels of nuclear TRAIL-R2 correlates with worse prognosis for PDAC patients suffering from early stage PDACs [19]. The level of TRAIL-R2 in the cytoplasm of PDAC cells, although significantly higher than in healthy tissue, did not correlate with any clinico-pathological parameter. To the best of our knowledge, no corresponding data for TRAIL-R1 are available so far. To fill this gap, we evaluated the clinical relevance of high levels of TRAIL-R1 in tumor tissues of PDAC patients with special emphasis to the possible differential impact of its cytoplasmic and nuclear localization.

## Methods

We retrieved formalin fixed and paraffin embedded PDAC and adjacent, peritumoral non-malignant tissue samples from the archives of the Institute of Pathology of the University Hospital Schleswig-Holstein and Christian-Albrechts-University Kiel, resected between 1999 and 2010. Follow-up data were retrieved from the Epidemiological Cancer Registry Schleswig–Holstein, Germany, hospital records and general practitioners. Patients were included if histology confirmed an adenocarcinoma of the pancreas. pTNM stage was determined according to the 8th edition of the Union for International Cancer Control (UICC) guidelines [22]. This study was approved by the local institutional review board of the Medical Faculty of the Christian-Albrechts-University of Kiel (A-110/99).

### Immunohistochemical staining

For immunohistochemistry 3 μm paraffin sections were deparaffinized in xylol and re-hydrated in a descending alcohol series. Antigen retrieval was achieved by heating for 15 min at 89 °C in citrate-buffer (pH 6.0). Intrinsic biotin and avidin binding sites were blocked with Avidin-Biotin Blocking Kit (Vector Laboratories, Burlingham, CA), endogenous peroxidase-activity with Hydrogen-Peroxide Block (15 min, RT; Thermo Scientific, Fremont, CA) and unspecific background was reduced with Ultra-Vision Block (5 min, RT; Thermo Scientific, Fremont, CA, USA). Slides were incubated with primary antibodies as previously described (clone TR1.02; Ganten et al., 2009 [23]). Bound antibodies were detected by a Super Sensitive IHC Detection System (BioGenex, San Ramon, USA). For color development, a Fast Red system (Sigma, Deisenhofen, Germany) was used. Washing steps were done with Tris-buffered saline supplemented with Tween (TBST). All slides were counterstained with hemalum and cover slipped.

### Microscopic evaluations and histopathological scoring

Evaluation of the staining was performed on a Leica DM 1000-Microscope (Leica, Wetzlar, Germany) and a two-dimensional scoring system was applied to semi-quantitatively assess the TRAIL-R1 expression data. The intensity of the staining was judged on an arbitrary scale of 0 to 3 with 0: no staining; 1: weak staining; 2: moderate staining and 3: strong staining. In samples with varying staining intensities, strongest values were stated. In addition, the percentage of stained cells was quantified and scaled from 0 to 4 with 0: no positive cells; 1: 1–10%; 2: 10–50%; 3: 51–80%; and 4: 81–100% positively stained cells. Both values were summarized in a sum score (Table 1) and separately assessed for cytoplasm and nuclei by two independent pathologists.

**Table 1:** Histomorphological evaluation score

Staining intensity	Points	Number of positive cells	Points
Negative	0	0%	0
Weak positive	1	< 10%	1
Moderate positive	2	10–50%	2
Strong positive	3	51–80%	3
–	–	81–100%	4

### Statistical analyses

Statistical analyses were done using SPSS 23.0 (SPSS, IBM Corporation, Armonk, NY, USA). For the correlation of the clinico-pathological patient characteristics and TRAIL-R1 expression, data were dichotomized and Kendall’s Tau (τ) was used. Only patients with existing follow-up data were included. Patients who died within 14 days after surgery and patients who received neoadjuvant treatment were excluded. Consequently, for these analyses 97 out of 106 patients were included. Out of these, 19 patients were censored because they were either alive or lost in follow up. The overall postoperative survival was analyzed. Staining intensities of tumor and normal tissues were compared using the Wilcoxon test as a nonparametric test for paired samples. Survival analyses were performed by Kaplan-Meier estimates and statistical evaluations were done by log-rank tests. *P* values ≤0.05 were considered significant.

## Results

### Patient collective

To investigate the significance of TRAIL-R1 for pancreatic cancer biology, we analyzed the staining intensity, the percentage of stained cells and the intracellular distribution of this receptor in sections of 106 tumors and 28 morphologically normal corresponding peritumoral ducts from 106 patients suffering from PDAC. Out of 106 patients, 51 (48.1%) were female and median age was 65 years (range 47–85 years). Cancer of the pancreatic head, corpus and tale were found in 75/106 (70.8%), 7/106 (6.6%) and 8/106 (7.5%) cases, respectively. In 16/106 cases (15.1%), the localization was not specified. The detailed clinico-pathological patient characteristics are summarized in Table 2. Most of the patients have undergone surgery at stage T3 (94/106; 88.7%) and had already developed lymph node metastases (84/106, 79.2%), whereas no patient was operated at stage T1. Resected tumors were well or moderately differentiated in 66.1% of the cases. In 89.6% (95/106), patients were free of distant metastases at the time of resection. Resection without residual tumor load was achieved in 74/106 patients (69.8%).

**Table 2:** Clinico-pathological patient characteristics on the basis of the TNM status (according to the UICC Classification of Malignant Tumors). Given are the total number of patients and the percentage (%)

Feature	n	%
T – category		
T1	0	0.0
T2	3	2.8
T3	94	88.7
T4	9	8.5
N - category		
N0	22	20.8
N1	84	79.2
NX	0	0.0
M - category		
M0	69	65.1
M1	11	10.4
MX	26	24.5
Venous invasion		
V0	79	74.5
V1	19	17.9
V2	3	2.8
VX	5	4.7
Perineural invasion		
Pn0	39	36.8
Pn1	59	55.7
PnX	8	7.5
Lymphatic invasion		
L0	30	28.3
L1	71	67.0
LX	5	4.7
R - status		
R0	74	69.8
R1	28	26.4
R2	2	1.9
RX	2	1.9
Histopathological grading		
G1	11	10.4
G2	59	55.7
G3	35	33.0
G4	1	0.9

T1: Tumor < 2 cm within pancreas; T2: > 2 cm, T3 over pancreas without infiltration of A. mes. Sup. or Truncus coeliacus, T4: vessel infiltration

### Expression of TRAIL-R1 in PDAC and non-malignant adjacent tissue

**Fig. 1:**
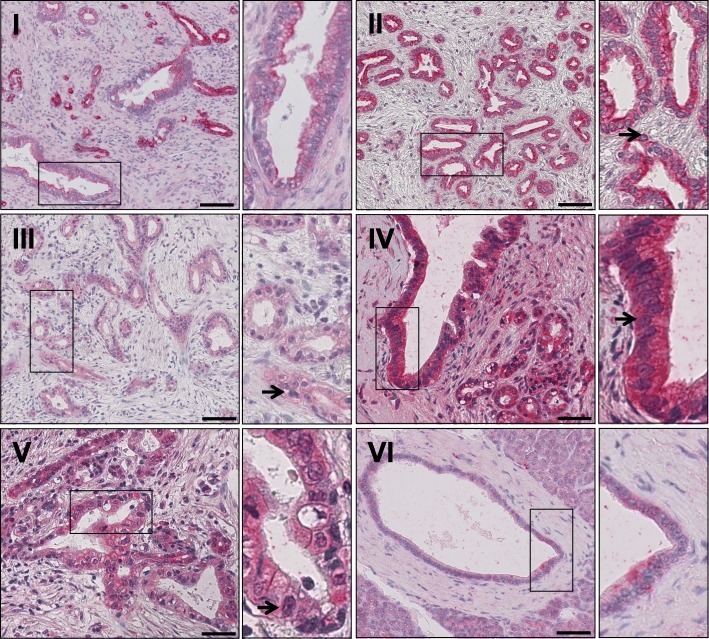
Representative images of TRAIL-R1 staining in PDAC tissue (I-V) and non-neoplastic pancreatic duct (VI). I: Tumors with strong cytoplasmic TRAIL-R1 staining in 51–80% cells and no nuclear TRAIL-R1 staining. II: Tumors with strong positive cytoplasm in > 80% cells with negative nuclear staining. III: Tumors with weak positive cytoplasmic staining intensity in > 80% of the cells. Weak positive staining in 10–50% of the nuclei. IV: Tumors with moderate positive cytoplasmic staining in > 80% cells. Weak positive nuclei in 51–80% cells. V: Tumors with moderate positive nuclei in 51–80% of the nuclei. Weak positive cytoplasmic staining in > 80% of the cells. VI: Non-neoplastic duct with weak to moderate positive cytoplasm staining and without positive nuclei. Magnifications corresponding to the rectangles in the large pictures are shown in the small windows. Arrows indicate exemplary nuclear staining. Scale bar marks 100 μm (I – III + VI) or 50 μm (IV – V)

TRAIL-R1 was expressed in 93.4% (99/106) of all PDAC samples (Table 3a). Analysis of its intracellular distribution revealed a cytoplasmic and nuclear localization, whereas plasma membrane staining was not distinct and therefore not evaluable. Representative images showing expression pattern of TRAIL-R1 in tumor tissue and non-malignant, adjacent tissue are shown in Fig. 1.

**Table 3:** Cytoplasmic and nuclear TRAIL-R1 expression in malignant and non-malignant ducts

	cytoplasm				nuclei			
a)	n		%		n		%	
Positive tumor cells								
0%	7		6.6		70		66.0	
< 10%	5		4.8		9		8.5	
11–50%	11		10.4		16		15.1	
51–80%	22		20.8		10		9.4	
> 80%	61		57.5		1		0.9	
Staining intensity								
Negative	7		6.6		70		66.0	
Weak positive	55		51.9		28		26.4	
Moderate positive	24		22.6		8		7.5	
Strong positive > 80%	20		18.9		0		0.0	
Sum score								
0	7		6.6		70		66.0	
2	4		3.8		9		8.5	
3–4	25		23.6		21		19.8	
5–6	57		53.8		6		5.7	
7	13		13.0		0		0.0	
b)	tumor		non-malignant		tumor		non-malignant	
	n	%	n	%	n	%	n	%
Staining intensity								
Negative	7	6.6	3	10.7	70	66.0	26	92.9
Weak positive	55	51.9	14	50.0	28	26.4	2	7.1
Moderate positive	24	22.6	6	21.5	8	7.5	0	0.0
Strong positive	20	18.9	5	17.9	0	0.0	0	0.0
Staining pattern: tumor vs. non-malignant	no difference ns, *p* = 0.698				*p* = 0.006			

a) number of positive cells, staining intensity and corresponding sum score are shown for cytoplasm and nucleus separately. Additionally, particular staining intensity in relation to histologic specification (tumor vs. peritumoral non-malignant) and for cytoplasmic vs. nuclear staining are provided (b)

In the cytoplasm, we found weak, moderate and strong TRAIL-R1 expression in 51.9, 22.6 and 18.9% (55/106; 24/106 and 20/106) of the cases, respectively (Table 3b). In 78.3% of the tumors, over 50% of the carcinoma cells showed positive cytoplasmic expression (Fig. 1). Cytoplasmic staining of TRAIL-R1 was present with very similar intensity in tumor and normal tissue.

In contrast, apparent differences in the nuclear TRAIL-R1 staining frequency and intensity were observed in tumor versus normal tissue (Table 3b, *p* = 0.006). Whereas overall 34% of tumors showed nuclear presence of TRAIL-R1 with either weak (26.4%; 28/106) or moderate (7.6%; 8/106) staining intensity, only 7.1% (2/28) of normal ducts expressed TRAIL-R1 in the nucleus and this with only weak intensity.

### Correlation of TRAIL-R1 expression with clinico-pathological parameters and patient survival

Next, we correlated the expression level of TRAIL-R1 and its intracellular distribution (cytoplasm and nucleus) with diverse clinico-pathological parameters like tumor stage (T), nodal spread (N), distant metastasis (M), grading (G), lymphatic invasion (L), venous invasion (V) and perineural invasion. As shown in Table 4, we found a significant negative correlation of the amount of cells positively stained for TRAIL-R1 in the cytoplasm with a tumor grading (τ = − 0.228; *p* = 0.043). Apart from that, no other correlation was found for cytoplasmic or nuclear staining with any of the parameters.

**Table 4:** Correlation of TRAIL-R1 expression with clinico-pathological parameters

Staining parameter	Tumor stage	Lymph nodes	Metastasis	Grading	Lymph vessels	Venous invasion	Perineural invasion
Intensity cytoplasm	τ = −0.018 *p* = 0.880	τ = 0.045 *p* = 0.703	τ = −0.096 *p* = 0.413	τ = −0.099 *p* = 0.382	τ = 0.127 *p* = 0.277	τ = 0.096 *p* = 0.404	τ = −0.033 *p* = 0.779
Number pos. cytoplasm	τ = − 0.115 *p* = 0.327	τ = − 0.080 *p* = 0.496	τ = 0.070 *p* = 0.548	τ = − 0.228 *p* = 0.043	τ = − 0.018 *p* = 0.877	τ = 0.044 *p* = 0.705	τ = 0.104 *p* = 0.375
Sum score cytoplasm	τ = 0.012 *p* = 0.918	τ = −0.006 *p* = 0.960	τ = −0.062 *p* = 0.598	τ = − 0.105 *p* = 0.351	τ = 0.071 *p* = 0.542	τ = 0.096 *p* = 0.407	τ = − 0.015 *p* = 0.896
Intensity nuclei	τ = − 0.210 *p* = 0.073	τ = − 0.006 *p* = 0.960	τ = 0.129 *p* = 0.272	τ = − 0.079 *p* = 0.482	τ = − 0.064 *p* = 0.584	τ = 0.096 *p* = 0.407	τ = 0.154 *p* = 0.188
Number pos. nuclei	τ = − 0.100 *p* = 0.392	τ = 0.172 *p* = 0.142	τ = 0.021 *p* = 0.858	τ = −0.057 *p* = 0.611	τ = − 0.005 *p* = 0.963	τ = 0.100 *p* = 0.385	τ = 0.148 *p* = 0.205
Sum score nuclei	τ = −0.028 *p* = 0.831	τ = − 0.010 *p* = 0.929	τ = 0.142 *p* = 0.224	τ = 0.079 *p* = 0.483	τ = 0.125 *p* = 0.285	τ = 0.134 *p* = 0.245	τ = 0.147 *p* = 0.210

*Abbreviations*: *τ* Kendall’s τ, *p p* value

Shown are correlation coefficients Kendall’s τ as well as the significance of the correlation

Further we explored whether the TRAIL-R1 expression pattern could be of prognostic value. To address this issue, we dichotomized the results for intensity and amount of positive cells as well as the sum score in a group with strong and in a group with weak expression of TRAIL-R1 (see Table 5) and analyzed these data by Kaplan-Meier analysis. Cumulative survival was compared by log rank test and *p* values ≤0.05 were considered significant.

**Table 5:** Impact of TRAIL-R1 expression pattern on survival of PDAC patient

Staining parameter	Total number of patients	Number of deceased patients	Median survival ± SD (95% CI) in months	*p* - value
Intensitiy cytoplasm				
Negative to weak positive	60	48	15 ± 3.611 (7.922–22.078)	
Moderate to strong positive	37	30	17 ± 3.513 (10.115–23.885)	0.735
Number of cells with positively stained cytoplasm				
≤ 80%	40	37	8 ± 3.041(2.041–13.959)	
> 80%	57	41	20 ± 3.855 (12.444–27.556)	0.004
Sum score cytoplasm				
≤ 5	65	52	15 ± 3.733 (7.682–22.318)	
> 5	32	26	17 ± 3.992 (9.177–24.823)	0.552
Staining intensity nuclei				
Negative to weak positive	89	70	15 ± 1.573 (11.918–18.082)	
Moderate to strong positive	8	8	13 ± 4.950 (3.298–22.702)	0.431
Number of cells with positively stained nucleus				
≤ 50%	88	69	15 ± 1.430 (12.198–17.802)	
> 50%	9	9	12 ± 4.472 (3.235–20.765)	0.687
Sum score nuclei				
≤ 3	19	17	8 ± 1.023 (5.995–10.005)	
> 3	78	61	16 ± 1.622 (12.821–19.179)	0.109

*Abbreviations*: *SD* standard deviation, *CI* confidence interval

*P*-values were estimated by log-rank-test with p ≤ 0.05 considered as significant

Neither the intensity of cytoplasmic staining nor the sum score showed a significant correlation with the patient survival. Likewise, nuclear staining showed no prognostic relevance.

**Fig. 2:**
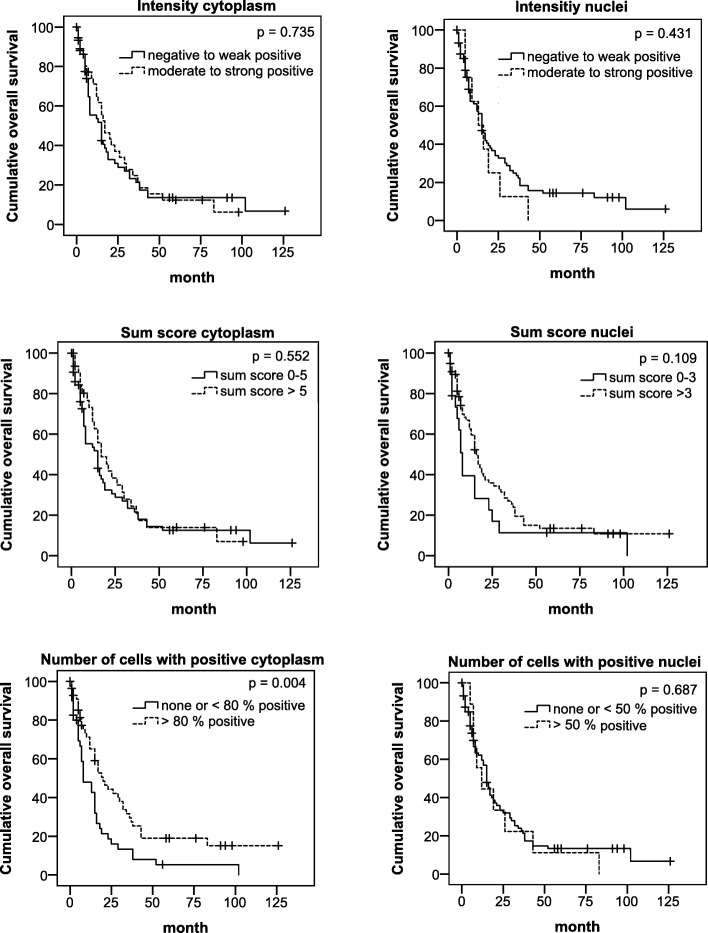
Kaplan-Meier analyses of the cumulative survival of patients with differential expression of TRAIL-R1. *P*-values were calculated by the log rank test and *p* ≤ 0.05 was considered significant

Since the immunostaining revealed differences in number of stained cells per tumor, we wondered whether this parameter could be of prognostic relevance for the patients. Importantly, we found that patients with tumors in which > 80% of the cells express TRAIL-R1 in the cytoplasm have significantly prolonged median survival compared to the patients whose tumors show TRAIL-R1 positivity in less than 80% cells (20 vs. 8 months; *p* = 0.004; Fig. 2).

## Discussion

Identification of the prognostic factors related to survival of cancer patients represents a strategy to understand the molecular mechanisms driving tumor progression and therapy resistance, and may consequently support the development of novel therapeutic strategies. The expression levels of TRAIL-R1 and TRAIL-R2 were shown to be of prognostic relevance for different tumor entities. In addition to their localization at the plasma membrane, TRAIL-R1 and TRAIL-R2 are also found in the cytoplasm and in the nucleus of several cell types. Especially in tumor cells, diminished plasma membrane but enhanced intracellular presence of these receptors was frequently observed. Importantly, despite these observations and emerging evidences for distinct compartment-specific functions of TRAIL-Rs, their cumulative expression levels - regardless of their intracellular distribution - are mainly taken into account when immunohistochemical studies are evaluated. However, recent studies on the TRAIL-R2-expression in PDAC tissues suggest the necessity of considering the intracellular distribution of TRAIL-Rs. Thus, the expression levels of TRAIL-R2 may emerge as either a positive or a negative prognostic marker, depending on the subcellular distribution (plasma membrane vs. nucleus) [19, 24].

Immunohistochemical studies described high cytoplasmic levels of TRAIL receptors in different cancer types, e.g. colorectal cancer [25–27], breast cancer cell lines [23, 28], renal cell carcinoma [29], NSCLC [30, 31], melanoma [32, 33], PDAC [19, 24], hepatocellular carcinoma [14], and glioblastoma multiforme [34]. Notably, the levels of just these intracellular receptors turned out to be of prognostic relevance in different tumor types [16]. Intriguingly, whereas high intracellular levels of TRAIL-R1 mainly correlated with positive patient’s prognosis, increased levels of TRAIL-R2 often correlated with shorter patient’s survival (for review [16]). These observations point to the existence of different, receptor-specific activities of cytoplasmic TRAIL death receptors and, in addition, suggest anti-tumor activities of intracellular TRAIL-R1, at least in some tumor entities.

In our present study, we identified cytoplasmic TRAIL-R1 as a positive prognostic marker for patients with PDAC. Interestingly, whereas overall staining intensity showed no prognostic relevance, the number of TRAIL-R1 positive cells per tumor turned out to be important for patient’s outcome. Noteworthy, we used an antibody for immunochemistry which is able to detect membrane expressed TRAIL-R1 as it has been shown before [23]. Specifically, patients with tumors in which more than 80% of cells showed cytoplasmic TRAIL-R1 staining had significantly prolonged survival compared to patients whose tumors presented with less than 80% cells positively stained for TRAIL-R1. In line with these findings, a significant negative correlation between number of cells with positive stained TRAIL-R1 in the cytoplasm and tumor grading was found. These results indicate that loss of cytoplasmic TRAIL-R1 may support recurrent disease with more malignant phenotype.

Little is known about the origin and sub-cytoplasmic localization of intracellular TRAIL death receptors. Cytoplasmic TRAIL death receptors have been detected in Golgi vesicles [32], endosomes [32] and autophagosomes [35]. In addition, their presence in soluble cytoplasmic fractions was also reported [19].

Likewise, the function(s) of cytoplasmic TRAIL receptors is still not fully understood. Sequestration of these receptors in autophagosomes could act as a strategy by which tumor cells escape TRAIL-induced apoptosis [17, 18]. On the other hand, internalization of TRAIL death receptors in response to TRAIL-treatment has been demonstrated, and may represent a part of TRAIL-induced signal transduction pathway [36]. Recently, the importance of cytoplasmic TRAIL-R1 in inducing cell death as a consequence of unresolved unfolded protein response (UPR) has been demonstrated [20, 21]. Noteworthy, in this case TRAIL-R1 mediated cell death is independent of TRAIL. Efficient UPR activation represents a characteristic feature of many human cancers. It allows the tumor cells to survive and adapt to adverse environmental conditions, promotes dormancy and also tumor growth, progression and therapy resistance. In this context, loss of cytoplasmic TRAIL-R1 would select cancer cells which are resistant to UPR-induced apoptosis and thus cells with more aggressive phenotype.

The TRAIL-TRAIL-R system has been shown to be of crucial importance in the tumor immune surveillance [12, 13]. Cytoplasmic TRAIL death receptors may represent a reservoir of receptors, which upon stimulation localize to the plasma membrane and boost the primary response of cells to TRAIL. Since PDAC cells preferentially utilize TRAIL-R1 to induce apoptosis in response to TRAIL, loss of TRAIL-R1 could lead to an escape of immune surveillance and accelerate the recurrent tumor growth.

Alternatively, it is also possible that cytoplasmic TRAIL-R1, via direct protein-protein interaction, sequesters TRAIL-R2 in the cytoplasm thus inhibiting its malignancy-promoting nuclear functions. According to this scenario, cells, which have lost cytoplasmic TRAIL-R1, would also present with a more aggressive phenotype.

Which, if any of these potential cytoplasmic TRAIL-R1 functions, accounts for the obviously anti-tumoral functions of cytoplasmic TRAIL-R1 remains to be elucidated. The existence of further, still unknown functions of intracellular TRAIL receptors is very likely.

Likewise, the cellular pathways leading to the observed loss of TRAIL-R1 in a subset of PDAC cells are not known yet. Recently, several mechanisms negatively regulating the cellular levels of TRAIL-R1, but not TRAIL-R2, have been registered in cancer cells. Thus, hypermethylation of TRAIL-R1 promotor leading to an epigenetic silencing of TRAIL-R1 gene was detected in ovarian cancer cells [37]. Furthermore, at the transcriptional level, negative regulation of TRAIL-R1 promotor by GLI3 as well as miR-25-dependent decrease of TRAIL-R1 levels were reported for cholangiocarcinoma cells [38, 39]. At the post-translational level, specific degradation of TRAIL-R1 protein was described in breast cancer and melanoma cells. Here, membrane-associated RING-CH (MARCH)-8 ubiquitin ligase targeted TRAIL-R1, but not TRAIL-R2, for lysosomal degradation. Interestingly, plasma levels of miR-25 are significantly elevated in PDAC and evaluation of the levels of miR-25 together with MIC-1 and CA19–9 was shown to be able to distinguish between PDAC, benign pancreatic disorders and other GI cancers [40].

## Conclusion

We show that cytoplasmic TRAIL-R1 may serve as a novel prognostic marker for PDAC patients. In addition, our data point to the necessity to investigate the evidently underestimated biological functions of intracellular TRAIL receptors.

## Abbreviations

CI: Confidence interval
GI: Gastrointestinal
IHC: Immunohistochemistry
PDAC: Pancreatic ductal adenocarcinoma
SD: Standard deviation
TRAIL: TNF-related apoptosis inducing ligand
UPR: Unfolded protein response
